# Application of 3D Bioprinting in Liver Diseases

**DOI:** 10.3390/mi14081648

**Published:** 2023-08-21

**Authors:** Wenhui Li, Zhaoyue Liu, Fengwei Tang, Hao Jiang, Zhengyuan Zhou, Xiuqing Hao, Jia Ming Zhang

**Affiliations:** 1 Department of Radiology, Yancheng Third People’s Hospital, Affiliated Hospital 6 of Nantong University, Yancheng 224000, China; 2 College of Mechanical and Electrical Engineering, Nanjing University of Aeronautics and Astronautics; Nanjing 210016, China; 3 Nanjing Hangdian Intelligent Manufacturing Technology Co., Ltd., Nanjing 210014, China; 4 Yangtze River Delta Intelligent Manufacturing Innovation Center, Nanjing 210014, China

**Keywords:** liver diseases, 3D bioprinting, biofabrication strategy, artificial multi-cellular tissues/organs

## Abstract

Liver diseases are the primary reason for morbidity and mortality in the world. Owing to a shortage of organ donors and postoperative immune rejection, patients routinely suffer from liver failure. Unlike 2D cell models, animal models, and organoids, 3D bioprinting can be successfully employed to print living tissues and organs that contain blood vessels, bone, and kidney, heart, and liver tissues and so on. 3D bioprinting is mainly classified into four types: inkjet 3D bioprinting, extrusion-based 3D bioprinting, laser-assisted bioprinting (LAB), and vat photopolymerization. Bioinks for 3D bioprinting are composed of hydrogels and cells. For liver 3D bioprinting, hepatic parenchymal cells (hepatocytes) and liver nonparenchymal cells (hepatic stellate cells, hepatic sinusoidal endothelial cells, and Kupffer cells) are commonly used. Compared to conventional scaffold-based approaches, marked by limited functionality and complexity, 3D bioprinting can achieve accurate cell settlement, a high resolution, and more efficient usage of biomaterials, better mimicking the complex microstructures of native tissues. This method will make contributions to disease modeling, drug discovery, and even regenerative medicine. However, the limitations and challenges of this method cannot be ignored. Limitation include the requirement of diverse fabrication technologies, observation of drug dynamic response under perfusion culture, the resolution to reproduce complex hepatic microenvironment, and so on. Despite this, 3D bioprinting is still a promising and innovative biofabrication strategy for the creation of artificial multi-cellular tissues/organs.

## 1. Introduction

The liver is a pivotal organ that balances biochemical environments and participates in various biochemical reactions in the human body. It is responsible for blood protein synthesis, glucose metabolism, and detoxification of metabolites [1]. Liver diseases are a major cause of morbidity and mortality across the globe; however, there is little to no progress in treatment options [2]. Liver cancer, a pivotal contributor to cancer mortality around the world [3], exhibits an obvious annual increase in occurrence rates [4], and it has been demonstrated to be the sixth most frequently diagnosed cancer and the fourth highest reason of cancer-caused death [5]. Currently, there are diverse risk factors that lead to liver cancer. These include bad diets, hepatitis B/C virus, alcohol, tobacco, smoking, obesity, and fatty liver disease [6,7]. Most patients with liver cancer are diagnosed at an advanced stage as a result of the lack of highly sensitive and effective detection tools [8]. The prognosis of patients at advanced stages is worse. Early-stage patients are eligible for surgical resection, transplantation, and ablative techniques, though they may suffer recurrence and metastasis owing to malignant tissue remaining in situ [9,10]. Patients at advanced stages could be treated with trans-arterial chemoembolization (TACE) and oral dosing with sorafenib. These patients always suffer from liver failure, and a shortage of organ donors or postoperative immune rejection are problems that remain to be solved [11,12]. High demand for liver transplantation exceeds the availability of suitable donor organs and, thus, the hope of patients with serious liver disease is small. New approaches for treating liver diseases, liver cancer included, are required.

From studies on NCBI, we see that researchers in this field are restricted by the lack of suitable models. Currently available models include 2D cell models, animal models, organoids, and Liver-Chip models. The testing of newly developed drugs in 2D monolayer cells and animals are time-consuming and expensive [13]. 2D cell models cannot truly mimic the real metabolic microenvironment of drugs and, thus, fail to reflect in vivo situations [14,15]. In addition, traditional animal experiments are uncontrollable and possess interspecific and metabolic differences, as well as suffering from ethical disputes [16]. Organoids present significant heterogeneity and reproducibility, but lack vascular, immunological, and stromal components, and the morphogenesis is poorly controlled during self-assembly process [17]. Liver-Chip models provide a more physiologically relevant environment when compared to traditional cell culture systems. These models usually consist of microfluidic devices that incorporate living liver cells and recreate the complex microarchitecture and functionality of the liver. This allows for better mimicry of the in vivo conditions, providing a more accurate representation of liver function and drug metabolism [18].

3D printing was first introduced as “stereolithography” by Charles W. Hull in 1986; this technique prints thin layers of a material in layers to form solid 3D structures during photochemical processes. The next generation was “3D bioprinting”, or additive manufacturing (AM) technology. Mironov et al. first proposed the concept of “3D bioprinting” in 2003 [19]. This technique aims to make contributions to tissue engineering and organ fabrication [20]. Currently, 3D bioprinting is the most promising technique for organ manufacturing. 3D bioprinting can be successfully employed to print living tissues and organs, containing blood vessel, skin, bone, cartilage, and kidney, heart, and liver tissue [21]. It has great potential in tissue and organ construction due to its precise control of the spatial distribution of cells and the surrounding microenvironment [22]. Usually, 3D bioprinting accurately set biologics in a layer-by-layer fashion in order to construct artificial multi-cellular tissues/organs [23,24]. 3D bioprinting can mainly be classified into inkjet 3D bioprinting, extrusion-based 3D bioprinting, laser-assisted bioprinting (LAB), vat photopolymerization, Freeform Reversible Embedding of Suspended Hydrogels (FRESH), and sacrificial printing. Materials, containing hydrogels and cells, are arranged for the 3D bioprinting of biological tissues and organs. 3D bioprinting is commonly employed for disease modeling, drug screening, and organ regeneration. In conclusion, although 3D bioprinting holds great promise for treatment of liver diseases, we still have much work to do prior to routine treatment by 3D bioprinting.

## 2. 3D Bioprinting Methods

**Figure 1:**
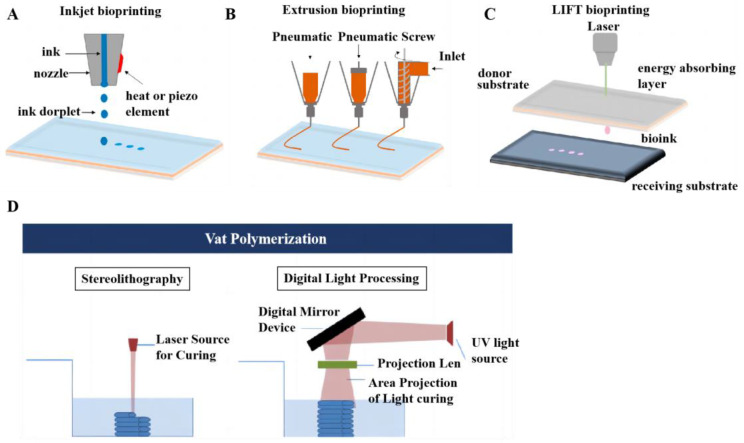
Four main techniques of 3D bioprinting. (**A**) Inkjet bioprinting. (**B**) Extrusion bioprinting. (**C**) LAB. (**A**–**C**) reproduced with permission [37]. (**D**) Vat Polymerization. Reproduced with permission [71].

Inkjet technology mainly includes two categories: continuous and drop-on-demand inkjet printings. Inkjet-based 3D bioprinting can generate droplets in the picoliter volume range, fire in a few seconds, and print in a noncontact manner [25], as demonstrated in Figure 1A. The inkjet technique can fabricate high-resolution structures with more precise modulation of the droplet size [26]. It has become a practical method in medicine in respect to scaffolding, cell deposition, and drug development. In 2003, Prof. Thomas Boland of Clemson University proposed the concept of “cellular inkjet bioprinting” and successfully realized live cell printing with PBS buffer containing Chinese hamster ovary cells (CHO) and mouse embryonic motor neuron cells as “bioink”, thereby laying the foundation for the development of cell inkjet bioprinting [27,28,29]. Arai et al. fabricated a 3D culture system by applying an artificial scaffold and inkjet 3D bioprinter for investigating liver-specific functions of hepatocytes through interaction of galactosylated alginate gel with asialoglycoprotein receptor [30]. Moya et al. also employed inkjet-based printing to construct electrochemical dissolved oxygen sensors along the microfluidic channel in liver-on-a-chip for monitoring oxygen concentrations [31]. 

Owing to its compatibility and ease of operation, extrusion-based 3D bioprinting is the most broadly applied technique for establishing scaffolds of liver tissue [32], as presented in Figure 1B. It is a cheap and easy-to-use manufacturing technique and is capable of producing precision medicines [33]. In extrusion-based 3D bioprinting, bioinks sustained a continuous layer-by-layer process to extrude filaments for production of 3D structures. The versatility of extrusion methodology introduces a novel avenue for generating biomimetic tissues and organs. Bouwmeester et al. generated porous constructs utilizing human hepatocyte-like cells gained from organoids upon extrusion-based printing to construct hepatic in vitro models [34]. Cuvellier et al. implemented a liver model via extrusion-based 3D bioprinting with hepatic cells, opening new perspectives in the molecular and cellular study of fibrosis [35].

LAB attaches the bioink to a layer of energy absorbing material and emits a laser to engender pulses, thereby ejecting the ink to the receiving platform below [36]. LAB can avoid direct contact between dispenser and bioinks without adding mechanical stress to cells, and thus cell viability is higher than 95% [37], as demonstrated in Figure 1C. Laser-assisted printing can also print highly viscous materials and use more types of bioinks. Touya et al. [38] offered a bone repair method through LAB-printed solidifying tricalcium silicate-based bioink. Nakielski et al. [39] elucidated LAB-generated injectable electrospun nanofibers that were highly biocompatible with bone and cartilage tissues, providing a mechanical environment.

Vat photopolymerization is the earliest and, relatively, the most mature type of 3D bioprinting. It employs the superposition molding of materials to establish several plane layers of a 3D target and scans the liquid photosensitive resin with a beam. Eventually, each layer is accumulated on the scaffold [40]. It makes stereolithography (SLA) and digital light processing (DLP). Vat photopolymerization outperforms other methods in relation to the speed and complexity of bioprinted structures, dimensional accuracy, and high surface quality, though it requires light-curing inks with photosensitivity and shear dilution, and material types are limited [41], as demonstrated in Figure 1D. In addition, there is no concern about cell damage resulting from nozzle blockage and shear force. Mahdavi et al. printed human corneal stroma equivalents using SLA [42]. Choi et al. established full-thickness wound models with DLP utilizing silk fibroin bioink [43].

FRESH is an embedded printing method that tackles this problem by extruding bioinks into a yield-stress support bath, which holds the bioinks in place until they are cured [44]. This technology is particularly well suited for the creation of high-fidelity complex tissue structures. It enables the creation of complex, fine, and structurally precise tissues. During the printing process, the support gel temporarily supports the bioink, minimizing distortion and maintaining the fidelity of the printed structure. This technique allows the creation of blood vessel-like channels, hollow structures, and complex tissue structures that closely resemble native tissues [45]. Eman et al. demonstrated the ability of FRESH bioprinting to produce patient-specific anatomical models using adjustable alginate bio-inks at full adult size [46].

Sacrificial printing has been widely used in recent years in the field of biotissue engineering and organ printing, which utilizes soluble support materials to create complex structures or achieve specific functions [47]. Sacrificial printing can be used to create bioprinted tissues with internal channels or vascular systems. In this case, the bioink material is printed as the main tissue structure while the soluble scaffold material is used to create the vascular network [48]. Compared to other 3D bioprinting methods, this type of printing becomes more demanding in terms of printing time and cost budget as sacrificial printing requires the preparation of additional scaffold structures and removal of scaffold materials. Hölken et al. synthesized novel 3D hollow aerospace silicon nano and microstructures using the sacrificial template method [49]. Cheng et al. investigated a strategy for the rapid preparation of bionic tissue models based on bacterial cellulose matrices using sacrificial 3D printing, further contributing to the potential scalability of sacrificial 3D printing technology [50].

These four technologies are the main categories of applied 3D bioprinting and their characteristics are summarized in Table 1. The data mentioned above are provided in order to offer a general understanding of the various techniques involved and may not represent exact values. The actual values may vary slightly depending on different experimental conditions and parameter settings.

**Table 1:** Characteristics of four 3D bioprinting techniques.

	Inkjet	Extrusion	LAB	Vat Photopolymerization	References
Speed	Fast	Slow	Medium	Fast	[51,52,53,54]
Cost	Low	Moderate	High	Low	[51,52,53,54]
Resolution	50 μm	100 μm	10 μm	1 μm	[55,56,57,58]
Cell viability	~80%	>90%	<85%	>85%	[59,60,61,62]
Cell density	<10^6^ cells/mL	Cell spheroids	<10^8^ cells/mL	10^8^ Cells/mL	[63,64,65,66]
Structural Integrity	Low	High	Low	High	[67,68,69,70]

## 3. Bioinks for 3D Bioprinting

### 3.1. Hydrogels

Bioinks are composed of a hydrogel pre-polymer solution and cells. Hydrogels, natural or synthetic polymer networks with high ability to absorb water, are the most suitable materials matching the mechanical, physical, and chemical properties with a natural extracellular matrix (ECM) [72,73]. Hydrogels, with printability, crosslinkability, biocompatibility, and mechanical properties, can directly provide structural support for cells to provoke differentiation, proliferation, and adhesion [74,75]. Printability means the relationship of bioinks with substrates, deciding the printing of accurate and high-quality patterns [76]. Printability is also affected by the crosslinkability of materials. Hydrogels should be in a liquid or a paste-like form, and the hydrogel pre-polymer solutions with controllable viscosity over a wide range are suitable in view of diverse cell densities and printing methods [77]. Biocompatibility refers to the appropriate host response of materials under specific condition, which requires harmless materials to cell proliferation that provide proper binding with cells for in vitro applications [78,79]. Applicable hydrogels can maintain sufficient mechanical properties, such as shear stress, strain, and compressive modulus post polymerization, in order to offer a stable environment to cells for proliferation, attachment, and differentiation [80].

Synthetic polymers are produced by chemical synthesis with more controllable chemical and mechanical characteristics [81,82]. Natural hydrogels contain polymers in ECM components such as collagen, fibronectin, gelatin, as well as others such as silk fibroin, chitosan, and alginate [83]. Alginate (most commonly used), collagen, decellularized ECM (dECM), and gelatin are employed in constructed liver models [84]. Alginate is isolated from seaweed, and it possesses the advantages of low cost, sufficient biocompatibility, and excellent formability [85]. Collagen is an ECM and an ideal natural biomaterial to encapsulate hepatocytes; however, it has poor mechanical strength, which causes the easy collapse of scaffolds [86]. Relative to natural and synthetic polymers with limited repair capacity, dECMs mimic a non-immune environment with native 3D structures and bioactive components [87]. Gelatin and relative derivatives are another category of hydrogel in liver 3D bioprinting [88]. Gelatin methacrylamide (GelMA), a photosensitive gelatin, is popular due to its excellent formability and biocompatibility. Hiller et al. describe the application of a bioink consisting of gelatin, alginate, and human ECM (hECM) in the printing of human HepaRG liver cells with a pneumatic extrusion printer [89]. Xu et al. fabricated a novel 3D breast tumor model through a bioink consisting of porcine liver-derived dECM with gelatin and sodium alginate [90]. Mazzocchi et al. designed a printable bioink by combining methacrylated collagen type I and thiolated hyaluronic acid to keep hepatocytes viable [91]. To maintain the shape and structure of collagen, Lee et al. applied polycaprolactone to build a framework and mixed collagen and cells in the canals [92]. Kim et al. developed a new dECM bio-ink by loading the dECM micro-particles into a gelatin compound, with enhanced 3D printability and mechanical properties [93]. In 2014, Pati et al. indicated that dECMs extracted from tissues were solubilized into bioinks for bioprinting [94]. Extrusion-based bioprinting is the most widely used method for constructing liver tissue scaffolds. Using extrusion-based bioprinting, a larger variety of materials with a wide range of viscosities can be constructed [95]. Extrusion-based bioprinting has advantages in terms of material printing flexibility and can be used to print major portions of liver units containing hepatocytes; when embedding microvascular systems is required, high-resolution digital light processing (DLP) technology would be a better choice to create complex scaffolds at a higher resolution. In addition, the experimental environment required for extrusion bioprinting is simple and easy to set up, and is sufficient for most application scenarios. Among them, to address the vascularization issue, coaxial extrusion bioprinting can be used to create large amounts of liver tissue with an adequate vascular system [96]. Digital Light Processing (DLP) bioprinting technology utilizes light irradiation to heal photosensitive hydrogels at specified locations. When compared to extrusion bioprinting, the digital light processing (DLP) bioprinting method has higher precision and allows for the construction of more complex structures [97]. However, this method requires a more complex laboratory environment and possible phototoxicity to cells in the construction of microstructures within the liver lobules using light-curing-based bioprinting [98]. Therefore, extrusion-based bioprinting is the dominant approach for the time being. Hydrogels applied for 3D bioprinting of livers from 2016 to 2022 are summarized in Table 2.

### 3.2. Cells

**Figure 2:**
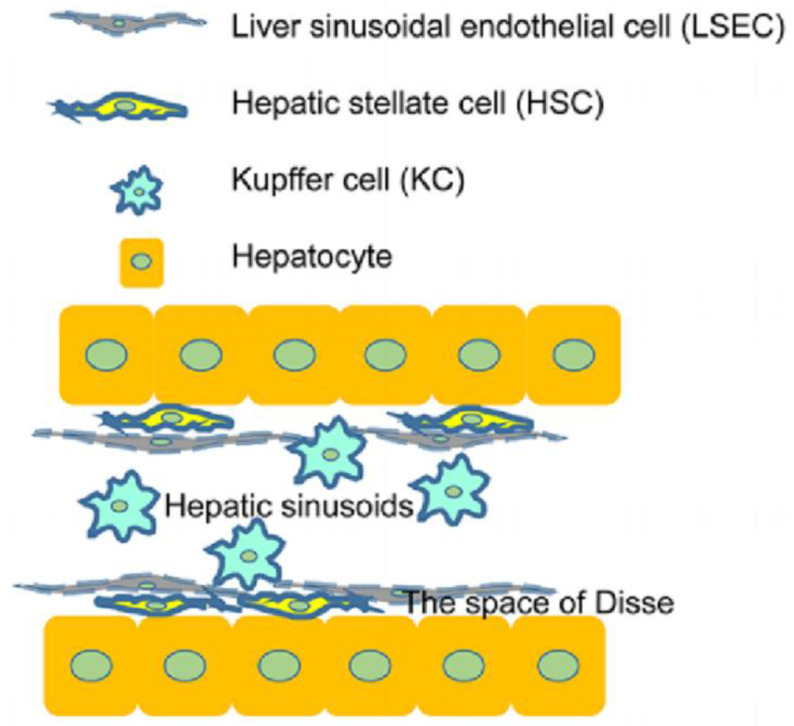
Main cell types of 3D bioprinting for livers. Reproduced with permission [125].

Cells are an innovative 3D-printed biomaterial for the manufacture of accurate cell models. Cells used for liver 3D bioprinting contain hepatic parenchymal cells (hepatocytes) and liver nonparenchymal cells (hepatic stellate cells, hepatic sinusoidal endothelial cells, and Kupffer cells) (Figure 2). Hepatic parenchymal cells exhibit the main functions of livers and nonparenchymal cells connect and support them. Primary hepatocytes, belonging to hepatic parenchymal cells, are the most desirable cell source for 3D bioprinting of liver tissues and are responsible for glucose metabolism and bile synthesis [112]. Nguyen et al. established a bioprinted liver tissue mimetic consisting of patient-derived hepatocytes and non-parenchymal cells [113]. However, human-sourced primary hepatocytes are deficient and are prone to lose phenotypes, and the culture is hard [114]. Hepatocyte-like cells differentiated from human adipose stem cells and hepatic cells derived from embryonic stem cells (ESCs) and human induced pluripotent stem cells (hiPSCs) are commonly used [115,116]. HepaRG cells, also hepatic progenitor cells, may be the most promising source in light of their potential for differentiation into hepatocytes in vitro and ability to form bile canaliculi [117]. Feng et al. introduced a versatile strategy to produce scaffolds from alginate and gelatin utilizing HepaRG cells and embryonic stem cells [118]. Under normal conditions, hepatic stellate cells are at resting state before they are then activated to increase collagen once the microenvironment is activated. Hepatic stellate cells were grown with parenchymal cells to imitate hepatic microenvironment and maintain phenotype and functions [113]. Ide et al. developed spheroids with primary human hepatocytes and hepatic stellate cells utilizing a 3D bio-printer [119]. Hepatic sinusoidal endothelial cells, directly contacting with blood flow, have high permeability and can eliminate soluble substances activated during inflammation [120]. Human umbilical vein endothelial cells (HUVECs), growing into capillary-like sprouts, can substitute hepatic sinusoidal endothelial cells for studies on vascularized livers [121]. Janani et al. bioprinted a human vascularized liver model by applying a liver ECM-based bioink laden with hepatic stellate cells, HUVECs, and adipose mesenchymal stem cell-derived hepatocyte-like cells using an extrusion-based bioprinting technique [101]. Kupffer cells are macrophages in liver sinusoids, the main functions of which are to extract particulates and toxins, mediate immune response, and to process and transmit antigens. Kupffer cells mainly process and transmit antigens, mediate immune response, and remove toxins and particulates in the portal vein [122,123]. Norona et al. added Kupffer cells to 3D bioprinted livers in order to test their effects on the injury/fibrogenic response under cytokine and drug stimuli, finding the importance of KCs in the fibrogenic response to agents [124]. Cell sources applied to the 3D bioprinting of livers from 2016 to 2022 are summarized in Table 2.

## 4. Applications of 3D Bioprinting

**Figure 3:**
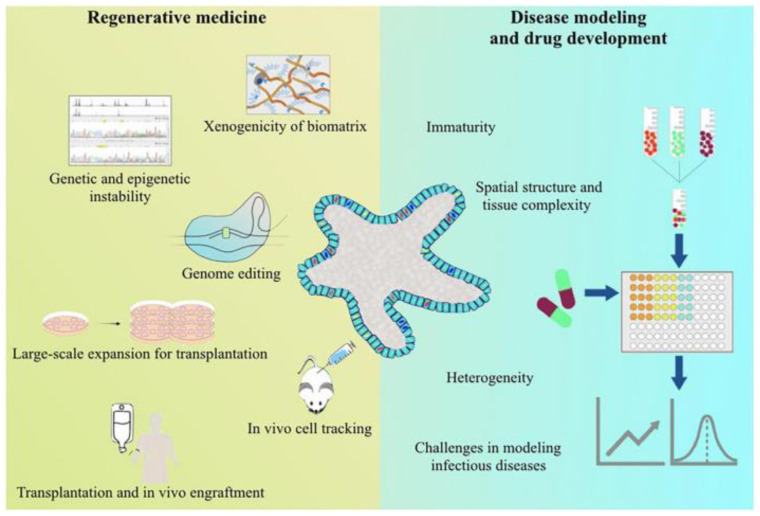
Direction of current 3D bioprinting application. 3D bioprinting was applied for disease modeling, drug screening and regenerative medicine. Reproduced with permission [145].

Over the past few decades, tissue engineering has made tremendous progress in fabricating tissue substitutes for clinical application. Relative to conventional scaffold-based approaches, which are limited in the production of constructs with functionality and complexity, 3D bioprinting allows accurate cell settlement, high-resolution, and biomaterials, better mimicking the complex microstructures of native tissues and precisely modulating cell distribution [126,127]. This technology is expected to make contributions to disease modeling, drug discovery, and even regenerative medicine [128,129,130] (Figure 3). First, livers are crucially important for drug metabolism and toxicity regulation. Bioprinted liver tissues can be used to produce human-relevant in vitro models in order to evaluate pharmacokinetics, toxicity, and efficacy, ultimately improving drug development and reducing animal testing [131]. Second, preclinical models accurately mimicking disease processes are vital for identifying new therapies. Bioprinted liver tissues can mimic liver fibrosis, hepatocellular carcinoma, and other conditions [132,133]. Third, bioprinted liver tissues can be utilized as a temporary scaffold to support the growth of new liver tissues, resulting in functional liver regeneration. They can also be utilized to create organoids for drug testing and personalized medicine [134,135]. Fourth, 3D printed liver tissue offers potentially important applications for organ regeneration and drug screening [136,137]. Three-dimensionally printed liver tissue could provide another option for patients who require organ transplants. This method can help patients recover their damaged liver function by converting the patient’s own cells into biomaterials and constructing complex vascular structures during the 3D printing process. Meanwhile, 3D printed liver models also have obvious superiority in drug screening. Since liver tissues for clinical applications must be three-dimensional in structure, the real human liver environment can be simulated using 3D printed liver models to more accurately assess key parameters such as drug absorption, distribution, metabolism, and excretion [138,139]. Finally, stable 3D bioprinted hepatocyte constructs would significantly facilitate drug testing for drug hepatotoxicity and liver injury in vitro [140,141]. Drug testing and disease modeling capabilities can be improved by integrating liver models and in silico simulations. For example, the exVive3D model, consisting of primary hepatocytes, hepatic stellate cells, and endothelial cells, is the first commercially available human liver tissue for assessing drug hepatotoxicity [142]. Liver organ printing is a promising technology in the field of regenerative medicine, but precise control of cellular spatial organization and functional maintenance, as well as the introduction of a functional vascular system in vitro, remain critical issues for disease modeling and regenerative therapy of the resulting liver-like organs [143,144].

**Figure 4:**
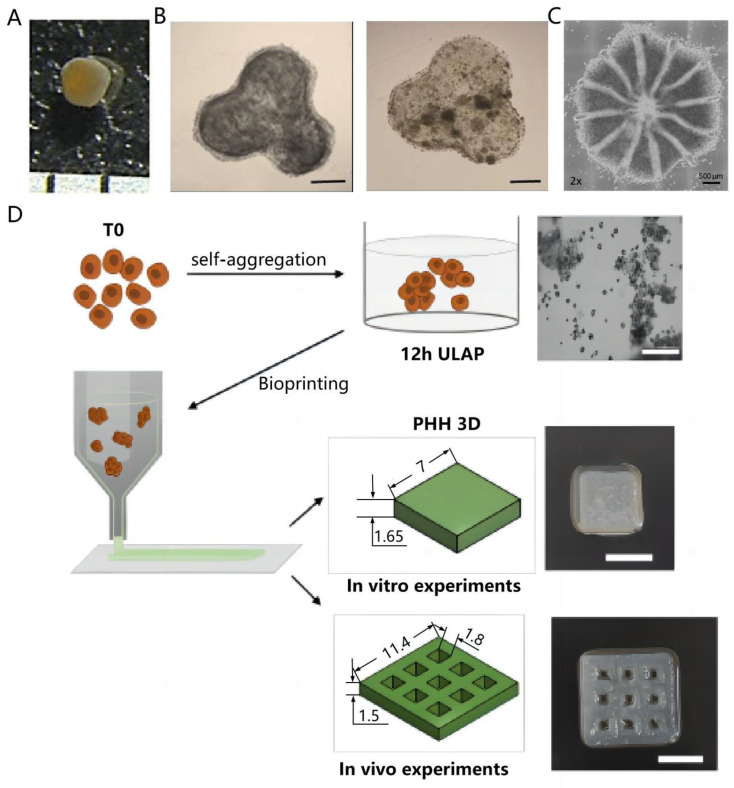
Applications of 3D bioprinting methods. (**A**) The roughly spherical printed liver tissue was shown after 60-d incubation, with the diameter about 1 mm. Reproduced with permission [149]. (**B**) Images for bioprinted hepatic constructs on day 7 and 14 (spheroid-like structures). Reproduced with permission [150]. (**C**) Cellbricks printing process of liver model [151]. (**D**) The bioprinting process of primary human hepatocytes in GelMa using an extrusion-based system. Reproduced with permission [153].

To study the biology and development of clinical products in liver disease, the 3D cell culture model is an indispensable tool [125]. In vitro models can mimic liver diseases, explain cell function affected by single and combinatorial microenvironmental cues, mitigate the risk of drug-caused liver injury, and enable cell-based therapies in clinical settings [146]. Combining organoids with organ-on-a-chip or 3D bioprinting can develop organoids and create models generalizing tissue or organ interactions [147]. An in vitro 3D cellular model is desirable for drug discovery and clinical applications, including patient-specific treatment [148]. Kizawa et al. applied a scaffold-free 3D bio-printing method to establish liver tissue for stably maintaining drugs as well as glucose and lipid metabolism [149] (Figure 4A). Moreover, Kang et al. established a hexagonal bioprinted hepatic fabrication with incorporation of the spinning condition with media stimuli, where enhanced proliferation, EMT and functionality of HepG2 cells, increased susceptibility to acetaminophen-induced hepatotoxicity, and a number of spheroids as well as hepatotoxicity prevention via *N*-acetylcysteine (NAC) were observed [150] (Figure 4B). This culture strategy is effective for recapitulating liver injury and repair, hence improving in vitro modeling for evaluating drug effect. Grix et al. accurately printed a complex liver-like organ using a light-curing forming bioprinting method [151]. Tremendous progress has been made in regenerative medicine in relation to the fabrication of functional tissue substitutes [152]. In 2022, Cuvellier et al. employed an extrusion-based system to bioprint primary human hepatocytes in a GelMA matrix, followed by organization into polarized hollow spheroids [153]. Bioprinted structures can vascularize and maintain hepatic specific functions for at least 28 d in mice after implantation, which suggests its promise for human liver tissue generation. Liu et al. fabricated soft vascularized tissue using multimaterial bioprinting upon a customized multistage-temperature-control printer, which formed 3D capillary networks, ensured cellular activities, and mimicked liver tissue with respect to synthesis of liver-specific proteins [154].

## 5. Discussion

The liver is a vital organ for metabolism and metabolic regulation. However, liver failure is still a major cause of mortality and the requirement for donor organs is increasing [155]. Faced with shortage of liver donors and postoperative immune rejection, great progress has been made in the use of 3D bioprinting in printing liver tissues. It has progressed over the past few decades as it can better mimic the complicated microstructures of tissues and precisely modulate cell distribution [156]. Commonly employed 2D monolayer cell cultures and animals for testing new drugs are expensive and time-consuming. These methods cannot reflect actual metabolic microenvironments of drugs in the human body, and metabolic differences of diverse species exist. Fortunately, 3D bioprinting strategies have developed into relatively mature strategies.

Presently, the main types of 3D bioprinting are inkjet 3D bioprinting, extrusion-based 3D bioprinting, LAB, vat photopolymerization, FRESH, and sacrificial printing. As mentioned above, these methods possess different operating principles and are used for various purposes of application. To achieve the bioprinting of liver units, many aspects must be considered. Extrusion-based bioprinting possesses better printing flexibility and can print the main part of livers with hepatocytes. To print the microvascular system, DLP technology can create complex scaffolds with higher resolution. Considering the cost-effectiveness, realizing the metabolism effects of hepatocytes is the priority for drug screening, and thus extrusion-based bioprinting is sufficient for most cases.

Different hydrogels and cells were applied as bioinks in diverse categories of 3D bioprinting. In the bioink, a biomaterial solution or a mixture of several biomaterials encapsulates cell types for the creation of tissue constructs, which are cross-linked or stabilized to produce the final shape and structure of the designed constructs [157]. An ideal bioink should have suitable mechanical and biological particularities in order to ensure correct functionality of bioprinted constructs. Usually, in constructed liver models, hydrogels containing alginate, collagen, dECM, and gelatin, as well as cells including hepatic parenchymal cells and liver nonparenchymal cells, are commonly employed. Hepatocytes are the main type of hepatic parenchymal cells. Liver nonparenchymal cells contain hepatic stellate cells, hepatic sinusoidal endothelial cells, and Kupffer cells.

The ability of 3D printing technology to create complex structures is an exciting development in tissue engineering. Nevertheless, ensuring the long-term structural and functional stability of printed tissues is critical to their success. Achieving high cell densities in printed structures is important for proper tissue function [158,159]. It is important to ensure that the cells are evenly distributed and can communicate with each other effectively. This can be challenging, as cells may undergo remodeling and rearrangement during culture. Minimizing deformation and maintaining the original structure of the printed tissue is essential to preserve its function. In addition, multicellular fidelity and functionality are key factors to consider for liver organ printing [160,161]. Different types of cells must be incorporated into the printed tissue in order to mimic the complexity of the liver organ. The patterning of cells and the extracellular matrix (ECM) in the printed structure plays an important role in determining the long-term outcome of the tissue. The liver is a highly complex organ, with multiple cell types working together to perform various functions [162]. By addressing issues such as cell density, deformation during remodeling, multicellular fidelity, and ECM templates, we can make significant advances in creating functional printed liver tissue.

3D bioprinting allows accurate cell settlement, high-resolution, and biomaterials for mimicking the microstructures of native tissues and modulating cell distribution [126,127]. This contributes to disease modeling, drug discovery, and regenerative medicine. Liver disease models are beneficial for the development of new drugs and reduce the failure rate of these developments. 3D-bioprinted liver constructs utilizing primary hepatocytes can produce the main structure and functions as well as accurately predict drug-stimulated hepatotoxicity. Lewis et al. employed gelatin to fabricate scaffolds with undifferentiated HUH7 cells and examined cellular functions to estimate the optimal scaffold structure [163]. The grid scaffold shape and pore size dramatically influenced hepatocellular cancer (HC) growth in vitro, which pushed researchers to change focus from shapes to hepatic functions. Ma et al. constructed a hexagonal hepatic lobule that contained hiPSCs and endodermal and mesodermal cells using DLP [164]. It formed a complicated liver microenvironment, representing sufficient HC functions. Paulina et al. succeeded in rapidly printing cell-filled structures and established a sterile perfusion chamber, turning the printed organoids into biofactories capable of modulating liver-specific ammonia detoxification functions according to the printed structures. The combination of ultrafast VBP processes with organoid technology has significant potential for advanced regenerative medicine approaches and in vitro model development for personalized drug screening and disease modeling [165]. Liver tissue constructs 3D-bioprinted by Nguyen et al., using patient-derived hepatocytes and nonparenchymal cells, were fabricated to assess the organ-level response to hepatotoxicity caused by a clinical drug [113]. Dose responses of Trovafloxacin and Levofloxacin reflected the response of this model to drug-induced liver injury. Yang et al. also created 3D-bioprinted hepatorganoids with in vivo hepatic function that could alleviate liver failure post transplantation. This hinted that 3D bioprinting could generate liver tissues for the transplantation of liver diseases.

## 6. Future Directions

Challenges in the application of 3D bioprinting cannot be ignored in relation to the stimulation of 3D bioprinting of in vivo liver microenvironments. First, the liver is a complex and heterogeneous organ, with multiple cell types and microstructures that require diverse fabrication technologies. Thus, we should combine different 3D bioprinting methods and other fabrication technics in order to reproduce liver functions in vitro. Second, for drug screening, static models are unable to reflect the dynamic response of drugs under perfusion culture. High throughput of 3D-bioprinted constructs will hamper their applications. Third, the resolution of 3D bioprinting is currently insufficient for the purposes of reproducing complex hepatic microenvironments. The scale of the printed hydrogel structure is too large to manipulate cells, and randomly distributed cells in the scaffold cannot ensure the subtle anisotropy. Moreover, subtle changes in oxygen and nutrient concentrations are uncontrollable in light of obtaining nutrients by soaking in the medium. Additionally, the cost of liver 3D bioprinting remains high and it requires precise design and manufacturing processes, which restricts its development and application worldwide. Despite these considerations, 3D bioprinting is still a promising and innovative biofabrication strategy for creating artificial multi-cellular tissues/organs, the innovation of which possesses the potential to revolutionize medical field and produce scaffolds for tissue and drug screening, organ transplantation, and even regenerative medicine [166].

Vasculature plays a crucial role in the liver’s function and is essential for maintaining proper tissue viability and functionality. When it comes to bioprinting liver tissue, one of the key considerations is the development of functional vasculature within the constructs. There are some approaches and challenges related to vasculature in bioprinted liver models.

Approaches for vasculature in bioprinted liver models include the follow methods: (1) Coaxial bioprinting: this approach involves using a coaxial nozzle system to simultaneously deposit bioink containing parenchymal cells and another bioink containing endothelial cells. This enables the creation of prevascularized tissue constructs with a functional vascular network. (2) Sacrificial materials: bioprinting can involve the use of sacrificial materials that serve as temporary templates for vessel formation. These materials can be printed alongside the cells and then removed, leaving behind hollow channels that can be lined with endothelial cells to create blood vessels. (3) Self-assembly: in this approach, endothelial cells and supporting cells are allowed to self-assemble into functional capillary-like structures. By providing appropriate culture conditions, the cells can form intricate networks, thereby mimicking natural angiogenesis.

Challenges in vasculature in bioprinted liver models include: (1) Vascular network complexity: peplicating the complex architecture and hierarchical organization of the liver’s vasculature is challenging. Bioprinting techniques must accurately recreate the varied vessel diameters, branching patterns, and connections found in the liver. (2) Integration with host vasculature: for successful implantation, bioprinted liver tissue must integrate with the host vasculature. Ensuring proper connection and functionality between the bioprinted vessels and the existing vascular network poses a challenge. (3) Scale-up and perfusion: as the size of the bioprinted liver tissue increases, achieving efficient perfusion becomes crucial. Proper perfusion is necessary to deliver nutrients and oxygen throughout the tissue construct and remove waste products.

Addressing these challenges in the vascularization of bioprinted liver models is essential for the development of functional liver tissue constructs that accurately mimic the native liver’s physiological and metabolic functions. Continued research and innovation in this area will contribute to the advancement of bioprinting technology for liver tissue engineering.

Faced with these challenges, liver 3D bioprinting can be improved in the following aspects: (1) Improvement of medical imaging technology. Improved imaging can offer higher quality raw data for liver 3D bioprinting. High-resolution medical imaging technique will better represent liver structure and lesion, thus enhancing the accuracy and quality of 3D-bioprinted models. (2) Selection of optimized materials. Materials mainly contain ceramics, metals, plastics, and other materials. Selected materials must be more suitable for reproducing liver structure and lesions, which further improves the simulation performance of 3D-bioprinted models. (3) Improvement of machine performance. Elevating the resolution and speed of 3D printers will substantially heighten its application effectiveness and cost-effectiveness. In the future, liver 3D bioprinting can provide novel methods for prevention and health management of liver diseases. 3D bioprinting models can prepare high-fidelity liver models for liver surgery training and education to improve surgical skills of doctors by simulating the real environment, thereby reducing surgical risks and failure rates. Relative to non-surgical methods, this new method, with better simulation and operability, will have more applications in liver medicine.

In the future, the combination of organoid and organ-on-chip technologies and 3D printing will offer great potential for several fields, including regenerative medicine, drug discovery, and personalized medicine. Micro organoid structures grown in the lab provide a more physiologically relevant model than traditional two-dimensional cell cultures. They can mimic the structure and function of human organs, enabling researchers to study disease mechanisms and test drug responses in a more accurate and personalized way [167]. Integrating 3D printing into organ-on-chip engineering allows for the creation of micro-organs with heterogeneity, ideal 3D cellular arrangements, tissue-specific functions, and even circulatory motions in microfluidic devices [168]. In addition, patient-specific organ models can be created using the patient’s own cells, enabling tailored drug screening and therapeutic optimization. The combination of organ tissues, organ-on-chip technology, and 3D printing paves the way for personalized medicine [169].

## 7. Conclusions

3D bioprinting techniques fabricate biomimetic tissues with the usage of biomaterials and living cells, in a specific pattern or on an existing 3D matrix. A bioartificial liver is one promising tool for liver diseases, regenerative medicine, and drug testing. Considering their adaptability to culture environments, high-resolution cell structures, production of 3D scaffolds for cell growth, inkjet printing, extrusion printing, LAB, and vat photopolymerization are commonly chosen. Greater efforts are still required to solve the limitations in replicating actual 3D liver tissue environment during disease modeling, drug discovery, and regenerative medicine.

## Figures and Tables

**Table 2:** Summary of hydrogels and cell sources in 3D bioprinting of livers from 2016 to 2022.

Cell Sources	Biomaterials	Techniques	References
HepG2 cells	Decellularized liver matrix, gelatin and polyethylene glycol	Extrusion	[99]
HepG2 and NIH/3T3 cells	Alginate, GelMa, cellulose nanocrystal	Extrusion	[100]
HepaRG cells	Gelatin	Extrusion	[11]
Huh7 and HepaRG cells	Methacrylated gelatin	Extrusion	[35]
Human adipose mesenchymal stem cell-derived hepatocyte-like cells, human umbilical vein endothelial cells, and human hepatic stellate cells	Liver ECM	Extrusion	[101]
Fibroblasts and hepatocytes	Alginate and methylcellulose	Extrusion	[102]
HepG2 cells, HUVECs and NHDFs	alginate and methyl-cellulose (algMC)	Extrusion	[103]
Huh7 cells	Decellularized liver matrix, silk fibroin, and gelatin	Extrusion	[103]
Human-induced pluripotent stem cells-derived hepatocytes	Gelatin/alginate	Extrusion	[104]
HepaRG cells	Alginate-gelatin	Extrusion	[105]
HepG2 cells	Alginate, gelatin	Extrusion	[106]
Human-induced pluripotent stem cell (hiPSC)-derived cardiomyocytes and hepatocytes	Liver decellularized extracellular matrix (dECM) bioink	DLP	[107]
HepaRG and human HSCs	Gelatin and PEG	DLP	[108]
human-induced hepatocytes	GelMA/dECM	DLP	[109]
human induced pluripotent stem cells (hiPSC)-hematopoietic progenitor cells (HPCs), human umbilical vein endothelial cells (HUVECs), and adipose-derived stem cells	GelMA	DLP	[110]
HUVECs and HepG2	Glycidal methacrylate-hyaluronic acid (GM-HA)	DLP	[111]

## Data Availability

Not applicable.
